# Transcatheter arterial embolization for hemothorax caused by spinal fracture without arterial injury: a case report and review of the literature

**DOI:** 10.1186/s13256-022-03568-4

**Published:** 2022-09-03

**Authors:** Naoki Matsunaga, Takuya Okada, Yuko Ono, Keigo Matsushiro, Koji Sasaki, Tomoyuki Gentsu, Eisuke Ueshima, Keitaro Sofue, Masato Yamaguchi, Koji Sugimoto, Takamichi Murakami

**Affiliations:** 1 Department of Diagnostic and Interventional Radiology, Kobe University Hospital, 7-5-2, Kusunoki-cho, Chuo-ku, Kobe, 650-0017 Japan; 2 Department of Disaster and Emergency Medicine, Kobe University Graduate School of Medicine, 7-5-2, Kusunoki-cho, Chuo-ku, Kobe, 650-0017 Japan

**Keywords:** Angiography, Computed tomography, Hemothorax, Spinal fractures, Transcatheter arterial embolization

## Abstract

**Background:**

Spinal fractures rarely cause hemothorax, and no treatment consensus has been reached. Conservative treatment is generally selected in cases without arterial injury, but there have been some reports of uncontrolled bleeding. Here we report a case of hemothorax caused by spinal fracture without arterial injury treated with transcatheter arterial embolization.

**Case presentation:**

An 88-year-old Japanese woman with back pain was diagnosed with hemothorax due to bleeding from an unstable fracture of the tenth thoracic vertebra. Contrast-enhanced computed tomography revealed no obvious arterial injury. We performed transcatheter arterial embolization of the bilateral tenth intercostal arteries to prevent rebleeding. The hemothorax did not worsen until surgical spinal fixation 9 days post-transcatheter arterial embolization, and she was discharged 30 days after admission.

**Conclusion:**

Transcatheter arterial embolization for hemothorax caused by spinal fractures without obvious arterial injury may be a useful bridge to spinal fixation.

## Introduction

Spinal fractures rarely cause hemothorax, accounting for 0.9% of all hemothorax cases [1]. A treatment strategy for hemothorax caused by spinal fractures has not been established. Transcatheter arterial embolization (TAE) has recently been performed in cases where contrast-enhanced computed tomography (CECT) showed an obvious intercostal or lumbar artery injury [2–4]. However, when no arterial injury or extravasation is noted by CECT, it is unclear whether conservative treatments are possible on the basis of a few unsuccessful reports [1, 4–8]. We report a case of TAE with successful outcomes for hemothorax caused by a spinal fracture without obvious arterial injury and extravasation. We also review published case reports of hemothorax caused by spinal fractures.

## Case report

**Fig. 1:**
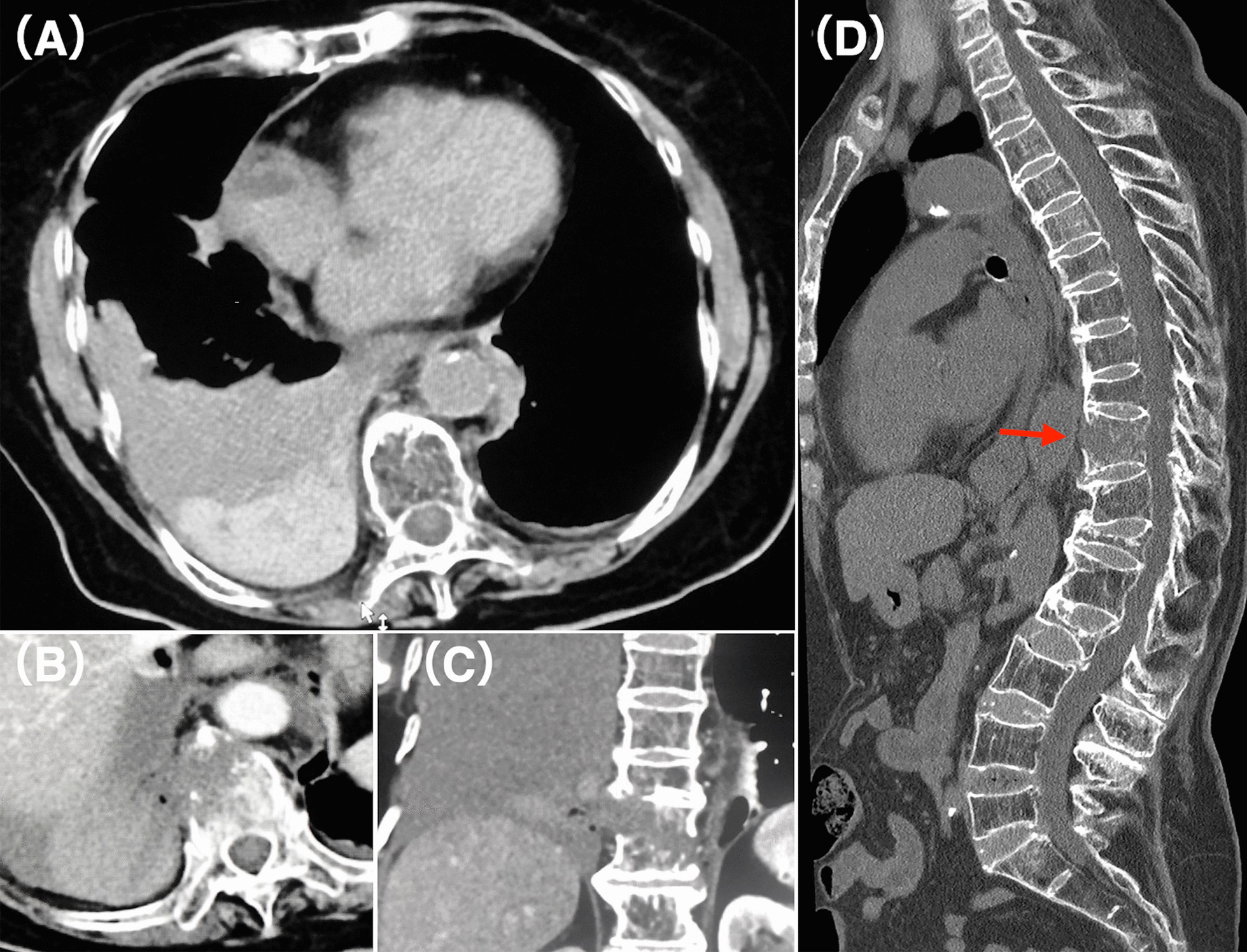
Contrast-enhanced computed tomography images. **A**–**C** Contrast-enhanced computed tomography images showing fracture of the tenth vertebra with a right hemothorax but without extravasation or arterial injury. **D** Sagittal bone image showing the reverse Chance-type vertebral fracture with wide separation of the anterior elements of the tenth vertebra (arrow) and anterior-flowing osteophytes from the eighth thoracic to the second lumbar vertebra

**Fig. 2:**
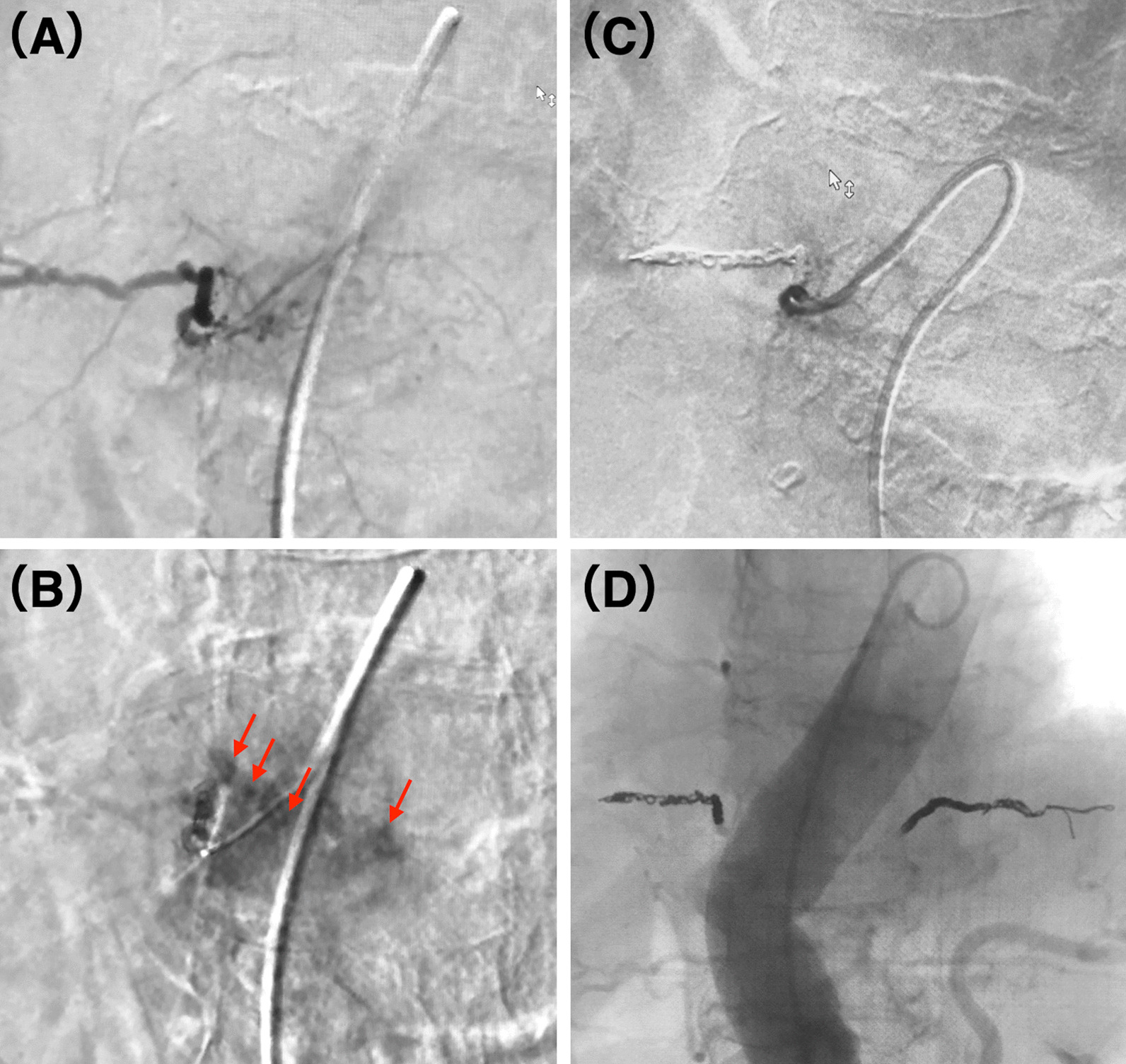
Angiography of the right tenth intercostal artery. **A** Selective angiography revealed no arterial injury, and no spinal branches were depicted. **B** Pooling of contrast medium (arrows) within the vertebral body was observed in the late phase. **C** Post-embolization angiography showing arterial occlusion and no contrast medium staining and pooling in the vertebra. **D** Digital angiography image taken after embolization of the bilateral tenth intercostal arteries

An 88-year-old Japanese woman with back pain and headache was admitted to our hospital, but the cause of injury was unclear. Her vital signs were stable (body temperature, 35.8°C; heart rate, 87 beats per minute; blood pressure, 180/90 mmHg; respiratory rate, 20 breaths per minute; percutaneous oxygen saturation, 100% on oxygen 2 L per minute via nasal cannula). She was oriented (Glasgow Coma Scale score, 15) but slightly agitated. CECT showed a massive right hemothorax, reverse Chance-type fracture of the tenth thoracic (T10) vertebra with wide separation of the anterior elements (Fig. 1), and mild traumatic subarachnoid hemorrhage. No obvious arterial injury or extravasation was observed. Computed tomography (CT) also revealed anterior-flowing osteophytes from the eighth thoracic (T8) to second lumbar (L2) vertebra causing diffuse idiopathic skeletal hyperostosis (DISH). The hemothorax was caused by bleeding from the fractured vertebra. We decided to perform angiography and TAE to prevent rebleeding owing to her inability to rest and thoracostomy to rule out arterial injury. A 5-French sheath was inserted into the right femoral artery, and aortography was performed using a 4-French pigtail catheter. The bilateral tenth intercostal arteries were selected with a 4-French shepherd hook catheter, and a 1.9-French microcatheter (Carnelian Si, Tokai Medical Products, Aichi, Japan) was introduced. Selective angiography revealed small pools of contrast medium in the T10 vertebra, but no arterial injury was observed. The radiculomedullary and anterior spinal arteries were not observed. After coil embolization of the tenth segmental arteries distal to the spinal branch with 0.014-inch pushable coils (C-Stopper, Piolax, Kanagawa, Japan), gelatin sponge particles (Serescue, Nippon Kayaku, Tokyo, Japan) were injected. The segmental arteries were then coil embolized as proximally as possible (Fig. 2). No complications occurred with these procedures. A chest tube was inserted on the same day as the embolization, and posterior fixation was performed 9 days later. During this time, the hemothorax did not rebleed. The patient was discharged 30 days after admission.

## Discussion

We demonstrated that TAE, a relatively safe procedure, may be an effective strategy to prevent hemothorax rebleeding until surgical spinal fixation is performed. Upon literature review of the PubMed and Ichushi (Japan Medical Abstracts Society) databases, we found 18 cases of hemothorax caused by spinal fracture [1–15], the clinical features of which, including our case, are presented in Table 1.

**Table 1:** Clinical features of 19 cases (including our case)

Author, year	Age (years)	Sex	DISH	Cause of injury	Fracture site	Fracture type	Arterial injury	Initial hemodynamics	Initial treatment for HTX	HTX exacerbation	Treatment for HTX exacerbation	Fixation (timing)	Prognosis
Singh, 2019 [15]	22	F	No	Traffic accident	T10–12	Burst	LA	Unstable	Stent graft	No	NA	Yes (day 4)	Survived
Hagiwara, 2009 [3]	25	M	No	Traffic accident	T9–11	Dislocation^†^	IA	Unstable	TAE (IA)	No	NA	Yes (NR)	Survived
Dalvie, 2000 [6]	28	M	No	Traffic accident	T4	Dislocation	None	NR	Conservative	Yes	Fixation	Yes (day 7)	Survived
van Raaij, 2000 [7]	55	F	No	Fall	T11	Chance	None	Unstable	Conservative	Yes	Thoracotomy	NR	Survived
Ninomiya, 2020 [1]	64	M	Yes^†^	Fall	T7, L1	Reverse Chance^†^	None	Stable	Conservative	Yes	Thoracotomy	Yes (day 10)	Survived
Matsushita, 2016 [10]	67	M	No	Hit by lumber	T3	Dislocation	IA	Unstable	TAE (IA)	No	NA	Yes (day 10)	Survived
Morita, 2009 [2]	68	M	NR	Fall	T11	Dislocation	IA	Unstable	Thoracotomy	No	NA	Yes (day 16)	Survived
Masteller, 2012 [9]	71	M	No	Transferred to bed	T11	Compression	None	Unstable	Conservative^‡^	No	NA	No	Dead
Lu, 2010 [11]	72	F	NR	Traffic accident	T11–12	Burst	None	Unstable	Thoracotomy	No	NA	Yes (day 6)	Survived
Hirota, 2019 [13]	74	F	Yes	Fall from standing	T11	Reverse Chance^†^	None	Unstable	Thoracotomy	No	NA	Yes (day 4)	Survived
Haruta, 2016 [12]	78	F	No	Traffic accident	T8	Reverse Chance	None	Unstable	Thoracotomy	No	NA	No	Dead
Okamoto, 2018 [4]	81	M	NR	Fall	T7	Reverse Chance^†^	None	Stable	Conservative	Yes	Thoracotomy	Yes (day 2)	Survived
Ninomiya, 2020 [1]	81	M	Yes^†^	Traffic accident	T8	Dislocation^†^	None	Unstable	Thoracotomy	No	NA	No^‡^	Survived
Okamoto, 2018 [4]	83	F	Yes^†^	Fall from standing	L1	Reverse Chance^†^	LA	Stable	Conservative	Yes	TAE (LA)	Yes (day 3)	Survived
Fukada, 2017 [5]	83	F	Yes^†^	Fall from standing	T12	Reverse Chance	None	Unstable	Conservative	Yes	Conservative	Yes (day 20)	Survived
Kaneko, 2000 [8]	86	F	No	Sit on a chair	T6	Dislocation	None	Unstable	Conservative	Yes	Thoracotomy	No	Dead
Okuda, 2021 [14]	92	F	Yes	Fall from standing	T12	Reverse Chance	IA	Stable	Conservative	Yes	Conservative	Yes (day 3)	Survived
Masteller, 2012 [9]	93	M	Yes^†^	Fall from standing	T10–11	Compression	None	Unstable	Conservative^‡^	No	NA	No	Dead
Our case	88	F	Yes	Unknown	T10	Reverse Chance	None	Stable	TAE	No	NA	Yes (day 9)	Survived

*F* female, *M* male, *DISH* diffuse idiopathic skeletal hyperostosis, *NR* not reported, *T* thoracic spine, *L* lumbar spine, *LA* lumbar artery, *IA* intercostal artery, *HTX* hemothorax, *NA* not applicable

^†^Diagnosed from the images in the paper. ^‡^No further treatment was desired.

Patients with DISH may suffer from hemothorax caused by spinal fracture. At least 8 of the 19 cases had DISH [1, 4, 5, 9, 13, 14], of which 6 were reverse Chance-type fractures on the basis of the descriptions and images [1, 4, 5, 13, 14]. DISH consists of ossification along the anterolateral aspect of at least four contiguous vertebrae and is common among the elderly. The most commonly affected area in patients with DISH is the lower thoracic spine and thoracolumbar junction, while reverse Chance-type fractures of the lower thoracic vertebrae occur by hyperextension due to mild trauma [16]. These events stretch and damage soft tissues including the pleura and are prone to causing complications of hemothorax.

Conservative treatment of hemothorax caused by spinal fractures is difficult. Six of the 19 patients had arterial injuries and required hemostatic procedures such as thoracotomy or TAE [2–4, 10, 14, 15]. Of the remaining 12 cases (excluding ours) that had no obvious arterial injury [1, 4–9, 11–13], four underwent emergency thoracotomy [1, 11–13], two died [9], and six received conservative treatment [1, 4–8]. However, all six conservatively treated patients developed worsening hemothorax; four underwent thoracotomy [1, 4, 7, 8], but one died [8]. In 13 cases, spinal fixation was eventually required [1–6, 10, 11, 13–15]. Spontaneous hemostasis was difficult owing to fracture instability and the lack of a compartmentalization effect resulting from insufficient paravertebral hematoma formation due to thoracic cavity perforation. Early stabilization is essential to control bleeding in hemothorax caused by spinal fractures [6]. However, temporary mechanical stabilization such as external fixation is impossible, and conservative treatments fail to control hemothorax until spinal fixation is performed.

**Fig. 3:**
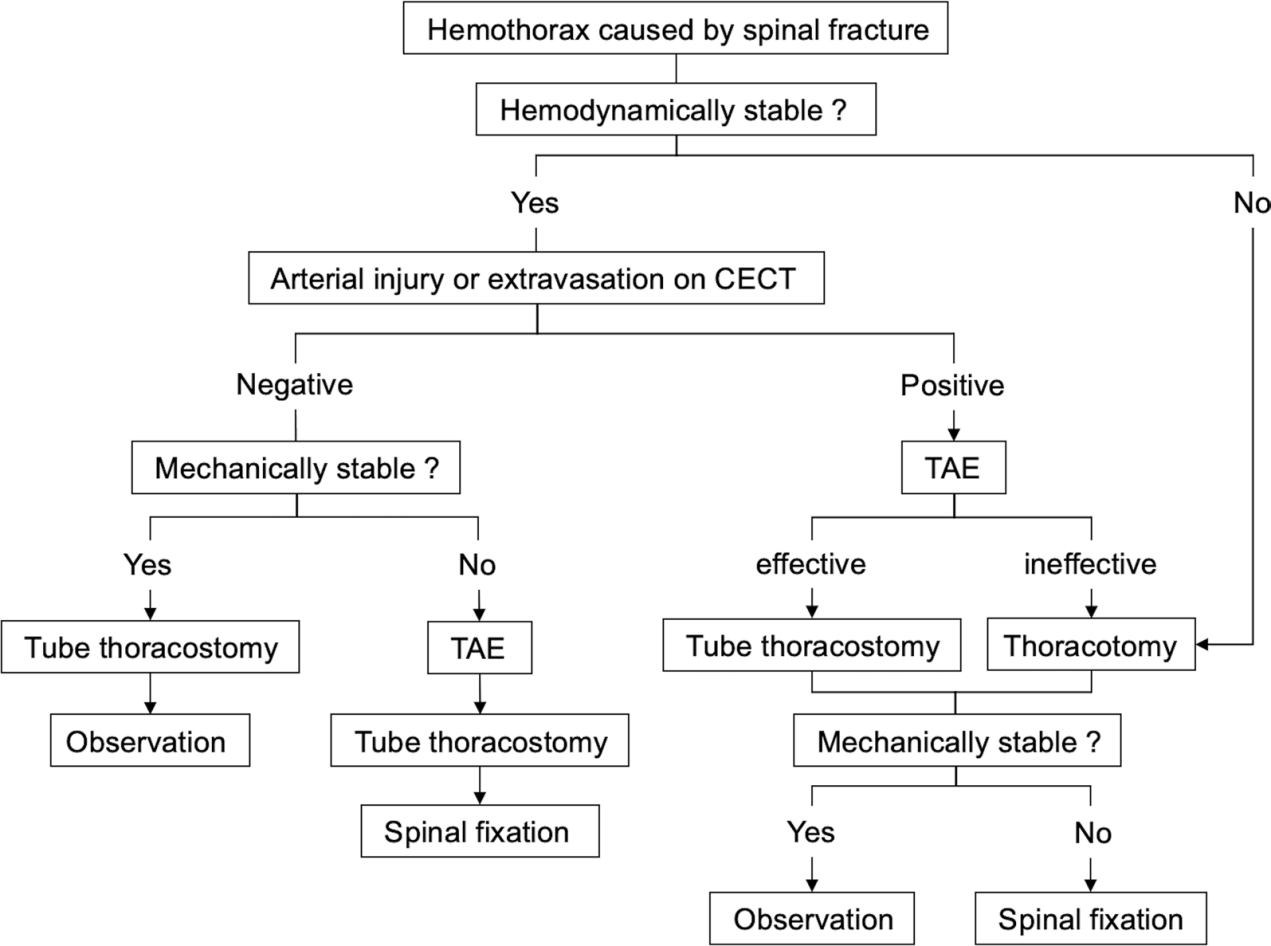
Management algorithm for hemothorax caused by spinal fracture. *CECT* contrast-enhanced computed tomography, *TAE* transcatheter arterial embolization

In vertebrectomy for spinal tumors, preoperative TAE reportedly reduced intraoperative bleeding [17]. TAE may also control bleeding from vertebral bodies in hemothorax caused by spinal fractures. In cases where extravasation within the vertebrae is difficult to identify by CECT owing to interference by the bone, angiography may help determine the diagnosis. Thus, we propose a management algorithm for hemothorax caused by spinal fractures (Fig. 3).

TAE is a relatively safe procedure for hemothorax caused by spinal fractures. Spinal cord ischemia is the most serious complication to be avoided in this procedure. The Adamkiewicz artery originates from the T7 to L2 vertebrae in 95% of cases [18], and is the preferred site for spinal fractures that cause hemothorax. The complication can be prevented by ensuring a lack of blood supply to the spinal cord by angiography before embolization and selection of appropriate embolic agents [17, 19]. However, a few cases of complications related to spinal cord ischemia due to embolization of unrecognized radiculomedullary arteries have also been reported [19]. Magnetic resonance imaging or CT angiography can identify > 90% of the Adamkiewicz artery [18]. There was one case report in which CT during angiography was reported as useful [20]. These diagnostic methods should be considered to prevent spinal complications.

## Conclusion

TAE was recently recognized as a good treatment option for arterial injury. However, it may also be an effective bridge to spinal fixation and/or an alternative to thoracotomy in case of hemothorax caused by spinal fractures in the absence of an obvious arterial injury.

## Data Availability

All data generated or analyzed during this study are included in this published article.

## Abbreviations

TAE: Transcatheter arterial embolization
CECT: Contrast-enhanced computed tomography
T: Thoracic spine
CT: Computed tomography
L: Lumbar spine
DISH: Diffuse idiopathic skeletal hyperostosis
